# Current Models of Investor State Dispute Settlement Are Bad for Health: The European Union Could Offer an Alternative: Comment on "The Trans-Pacific Partnership: Is It Everything We Feared for Health?"

**DOI:** 10.15171/ijhpm.2016.116

**Published:** 2016-08-20

**Authors:** Martin McKee, David Stuckler

**Affiliations:** ^1^ECOHOST, Department of Health Services Research and Policy, London School of Hygiene and Tropical Medicine, London, UK.; ^2^Department of Sociology, University of Oxford, Oxford, UK.

**Keywords:** Social Determinants of Health (SDH), Trade and Investment Policy, Population Health, Global Governance for Healt

## Abstract

In this commentary, we endorse concerns about the health impact of the trans-pacific partnership (TPP), paying particular attention to its mechanisms for investor state dispute settlement. We then describe the different, judge-led approach being advocated by the European Commission team negotiating the Trans-Atlantic Trade and Investment Partnership, arguing that, while not perfect, it offers significant advantages.

The difference between individual and public health is often illustrated by the classic tale of two people on the bank of a river down which drowning bodies are floating. One person, representing individual clinicians, works frantically to pull the bodies to safety. The other person, representing public health professionals, instead runs rapidly upstream to stop the crazed individual pushing their victims off the bridge.

Yet, increasingly, we recognise that the cause of this suffering is not a single individual on a bridge. Instead, at the source of the river, we can see the headquarters of large multi-national corporations using ever more sophisticated tactics to lure victims to their fate. Public health professionals who have the audacity to suggest that the bridges from which they are falling might be protected with guardrails are ridiculed for promoting a “nanny state.” In other words, we have discovered the commercial determinants of health.^1^

These powerful multi-national corporations drive global patterns of disease.^2^ Some, such as the tobacco and arms industries, profit from sales of products that kill people directly. Others, such as the food and sugar sweetened beverage industries, have a less direct impact, using combinations of pricing, marketing, and distribution to replace traditional nutritious foods.^3^ And finally there are those whose goal is to improve health, such as the pharmaceutical industry, but which adopts strategies that maximise their profits, even though this may deny life-saving medicines to those in most need.^4^

Several decades of corporate consolidation and concentration, driven by ever more mergers and acquisitions, mean that many of these corporate actors are now far larger, and much more powerful than individual countries. In the case of food, just 5 companies control over 40% of world food trade.^5^ These powerful corporations can, in effect, dictate policy to elected governments, for example by threatening to withhold foreign investment. Unlike smaller domestic companies, these multi-nationals can extract concessions on taxation, government subsidies, including investment in the infrastructure to serve their needs,^6^ and where these fail, they can employ mechanisms such as transfer pricing^7^ to ensure that their profits are banked in a low tax jurisdiction and thus, they can avoid giving anything back to the countries in which they are operating. These corporations are able to extract these rents because governments allow them to, and the power of labour to keep these forces in check has been weakened.

As the accompanying paper by Labonté and colleagues point out, governments negotiate regional and global trade deals that, while ostensibly removing barriers to trade, such as tariffs and non-tariff barriers, have much more to do with creating a market environment favourable to large companies. In effect these trade agreements transfer enormous power to multi-national corporations. Harmonisation of standards in areas such as environmental protection and health and safety has lowered them to the lowest common denominator. They have adopted and enforced intellectual property regimes that confer ownership of knowledge, much of it generated with public funding, on private corporations.^8^

The public health community has, in many cases belatedly, recognised the importance of these agreements, drawing attention to their secrecy and their often damaging effects on health, for example by demonstrating empirically the strong link between trade liberalisation and consumption of health damaging products.^9^

Labonté and colleagues, who have pioneered research on the health effects of international trade deals, make an important contribution by analysing in detail the health impact of one of the most controversial of these agreements, the trans-pacific partnership (TPP).^10^ They identify five elements of this agreement that give cause for concern: changes to intellectual property rights, sanitary and phytosanitary measures, technical barriers to trade, investor state dispute settlement processes, and regulatory coherence on issues such as health systems and policy. If the TPP is actually ratified then, as they note, there are potentially severe implications for health. However, in their final paragraph they also inject a note of caution. The TPP might never be ratified by the US government. At the time of writing, President Obama is making a final effort to get it through Congress before the end of his term in office, knowing that both main presidential candidates are opposed to it.

The TPP is, however, only one of a number of major trade deals currently being negotiated. Another, which has also attracted widespread attention, is the Transatlantic Trade and Investment Partnership (TTIP). This seeks to reduce barriers to trade between the United States and the European Union (EU) and has many features that are similar to the TPP. And, as with the TPP, its future is now uncertain, with growing opposition in both Europe and the United States, including on grounds of public health.^11,12^

The TTIP is, however, different in at least one way that has implications for international trade agreements more generally. As Labonté and colleagues note, the public health community has expressed great concern about existing investor state dispute settlement processes. These enable private companies to seek damages from governments whose policies appropriate their property or reduce their current and future income. They have increased markedly in number in the past decade, with many evolving challenges to health or environmental protection. The resolution process is secretive and expensive, and there is little opportunity to inject consideration of public policies beyond trade, such as the promotion of health. Unsurprisingly, it is an approach favoured by corporations.

The current investor state dispute settlement process is rigged in favour of multi-national corporations, for two reasons. First, it advantages those with the greatest resources. For many small countries, the cost of defending their position can be prohibitive. In a recent landmark case, Uruguay did manage to defeat a challenge to its tobacco control policies, but only with considerable financial support from the philanthropist Michael Bloomberg.^13^ On several occasions, the government considered conceding because of the escalating costs. As has been noted elsewhere, the very existence of this process has a chilling effect on governments seeking to promote health.^14^

Second, it provides a setting in which corporations often win. This is in marked contrast to the courts that ordinary citizens depend upon. In a systematic review of litigation, frequently involving constitutional law, initiated by the tobacco industry against governments, we showed that the industry almost always fails and, on the rare occasions when it succeeds initially, the courts provide clear guidance on how the law can be clarified to ensure that the government policy is ultimately sustained.^15^

These concerns explain why public health advocates in Europe have focused their attention on the investor state dispute settlement process in the TTIP. Importantly, they have achieved some success. The EU’s negotiating position argues for a number of important safeguards. One is the exclusion of the health sector from competition clauses, recognising that markets for healthcare are rife with failures. For example, European governments will not be forced to concede their power to maintain monopolies in the provision of health services, which is especially important to National Health Systems in the United Kingdom, Spain, and Italy, among others. The EU also insists on being able to provide subsidies to healthcare service providers, including selective subsidies to those based within the EU. And they will retain the right to regulate essential public services, for example by setting quality standards or accreditation mechanisms.

Perhaps most interesting is the ISDS process itself. Unlike the traditional approach to ISDS, in which decisions are made by arbitrators, the EU is insisting that the process should be overseen by publicly appointed, legally qualified judges. These should be selected at random from a qualified pool and should be free of conflicts of interest, which is not always the case at present. The grounds for seeking resolution of the dispute would be much more narrowly defined than at present, for example by being limited to discrimination on grounds of gender, race, religion, or nationality. All proceedings should be transparent, with documentation publicly available. Crucially, all parties with the legitimate interest in the dispute, which would include advocates for health, environmental protection, or human rights, would have a right to intervene. In these ways, the EU is proposing a radically different way of resolving international trade disputes. It is not, however, at all clear whether these will be acceptable to the United States and, as a number of European governments and the European Parliament have made clear that these are lines in the sand, it is far from certain that the TTIP will ever be ratified.

Yet, whether the EU will get what it wants in the negotiations is unclear. The Danish physicist Nils Bohr famously commented that “prediction is difficult, especially about the future.” Since the paper by Labonté and colleagues was published, the citizens of the United Kingdom have, inexplicably, voted to leave the EU. Whether the British government ever succeeds in doing so is itself far from certain, given dawning recognition of the enormous complexity of the task and the growing evidence that the ministers charged with extracting the country from the EU have only the sketchiest understanding of either European structures or international trade. However, among the many mutually contradictory and rapidly changing policies being advocated by British politicians, it is clear that there is an unwillingness to remain subject to rulings by the European Court of Justice. This, it must be remembered, is the court that has consistently observed the European Treaty obligation that the highest standard of health must be included in all EU policies. Instead, several of the politicians seem to be advocating a relationship with the EU based on World Trade Organisation (WTO) provisions. This position appeared briefly in a somewhat confused press release posted fleetingly by the UK’s newly created Department for International Trade that was rapidly withdrawn as “an error.” Such an approach would lack any of these aforementioned protections.

Ironically, the more populist advocates of leaving the EU invoked the TTIP, which they presented as a vehicle for corporate influence, as a reason for voting to leave. Yet, if they ever managed to negotiate a separate free-trade deal with the United States, a goal that seems vanishingly unlikely given the complete absence of British trade negotiators, it is almost certain that it too will lack any of the protections being insisted upon by the EU in its transatlantic dealings.^16^

In this commentary, we have looked at the other major trade deal currently being negotiated and show that, in at least one respect, the way that it addresses disputes between governments and corporations, there is an alternative approach. This will not solve all of the problems associated with TTIP, by any means, but it does show that there are some things that can be done to tackle some of its worst features.

Labonté and colleagues have done much more than comment on the threats posed by the TPP. They have also provided a very useful template against which all future trade deals should be evaluated. They have offered a new approach to confronting those crazed individuals at the source of the river who threaten the public’s health. Clearly, developing new tools and approaches for responding to the commercial determinants of health is a topic that will continue to attract the attention of public health researchers and advocates for many years to come.

## Ethical issues

Not applicable.

## Competing interests

Authors declare that they have no competing interests.

## Authors’ contributions

Both authors contributed equally to the writing of this paper.

## Authors’ affiliations

^1^ECOHOST, Department of Health Services Research and Policy, London School of Hygiene and Tropical Medicine, London, UK. ^2^Department of Sociology, University of Oxford, Oxford, UK.
